# An Account of a Monster of the Human Species, in Two Letters; One from Baron Reichel to Sir Joseph Banks, Bart., and the Other from Mr. James Anderson to Baron Reichel

**Published:** 1789

**Authors:** 


VI. *An Account of a Monſter of the human Species, in two Letters ; one from Baron* Reichel *to Sir* Joſeph Banks, *Bart., and the other from Mr.* James Anderſon *to Baron* Reichel. Vide *Philoſophical Tranſactions of the Royal Society of London*, Vol. LXXIX. for the Year 1789, Part II. 4to. *London*, 1789.

THE extraordinary ſubject of this account is a Gentoo boy, named Peruntaloo, who was born at Popelpahdoo, ſeventy miles weſt from from Mufilipatnam, and who, at the time* the two letters are dated, was living at Fort Saint George. He was then thirteen years old, and meafured 4 feet 6½ inches in height.

He is faid, by Baron Reichel, to poffefs a fhare of underftanding fuperior to that of the generality of boys of his age; and is handfome and well made, if we except the præternatural appendage that conftitutes the monftrofity. This appendage confifts in the loins and lower extremities of a male fufpended by the os pubis; an elongation of the xiphoid cartilage of Peruntaloo having anaftomofed with the fymphyfis pubis of the femi-monfter.

Baron Reichel remarks, that about the lower part of the loins of the femi-monfter he obferved an internal motion rather more confpicuous than in any other part of the body; and that upon queftioning Peruntaloo, he fhewed him that, by retaining his breath, he could force a current of air into them, fo as to fwell the parts like two blown up bladders, with a rumbling noife at the time of action. The Baron adds alfo, that the erection of the penis in the femi-monfter, and the command Perun-

* February, 1788.

taloo taloo has of difcharging the urine through it, are facts perfectly afcertained.

Mr. Anderfon, in a fhort anatomical defcription of the monfter, obferves, that the lower orifice of the ftomach feems to lie in the fac or cylindrical cavity between the two brothers on the right fide, and what may be reckoned the right hypochondre of the little one, as that part is tumid and full after eating; that the alimentary canal muft be common to both, as the anus of the little one is imperforate; that there is a bladder of urine diftinctly perceived, which occupies the left fide of the fac, or left hypochondre of the monfter; and that befides thefe there remain only the facrum, offa innominata, and lower extremities, perfect.

Mr. Anderfon adds, that Peruntaloo fays he has as complete a fenfe of feeling with every part of the body of his little brother (for fo he ftiles the femi-monfter) as of his own proper body; and this, Mr. Anderfon thinks, may account for the erection of the penis of the femi-monfter, and for the power Peruntaloo has of making water through it; but this volition, Mr. Anderfon obferves, does not extend to the legs or arms, which are cold in comparifon with the reft.

Two

Two engravings, from drawings made by Baron Reichel, illuſtrative of the ſubject, are given in the volume before us, to which we muſt refer our readers.

The account given of this monſter reminds us of the "frater pectori fratris adnatus," deſcribed and delineated by Bartholin *; and of another

* "Lazarum Colloredo Genuenſem Hafniæ primum vidi, "deinde Baſileæ, 28 annos natum, ſed utrobique cum ſtu-"pore. Fraterculus huic Lazaro in pectore erat adnatus, ſi "rectè conjeci, oſſe xyphoide utriuſque cohærente. Pes "ſiniſter ſolus illi dependebat, duo brachia, tres in manibus "ſingulis tantum digiti. Veſtigia pudendarum partium com-"parebant. Manus, aures, labia movebat, in thorace pul-"ſus. Excrementa nulla minor frater excernit niſi per os, "nares, et aures, nutriturque eo quod major aſſumit. Unde "partes animales et vitales diſtinctas habebit, quum et dor-"miat, ſudet, moveatur, quando major vel vigilet, vel qui-"eſcat, vel ſiccus eſt. Uterque etiam ſuo nomine ad baptiſ-"matis fontem inſignitus fuit, major Lazari, minor Joannis "Baptiſtæ. Naturalia vero viſcera ut hepar, lien, &c. utri-"que communia erant. Oculi clauſi fere Joanni Baptiſtæ, "reſpiratio minor, admota enim plumâ parum movebatur, "admotâ verò manu exilem halitum calentem deprehendi-"mus. Patulum ferè illi et hians os, dentibus prominulis, "ſaliva perpetuò ferè madens. Caput videbatur ſolum omne "alimentum in ſui augmentum abſumere. Prægrande enim "et majus quam Lazaro, ſed deforme, capillis ſupino ſitu "dependentibus. Barba utrique crevit, ſed Baptiſtæ neglecta, "Lazaro another monſter of the ſame kind, of which an account *, accompanied alſo with a figure, is given in the works of Ambroſe Paré.

